# What is Currently Known about Odontogenic Keratocysts?

**DOI:** 10.3290/j.ohpd.b3240829

**Published:** 2022-07-22

**Authors:** Andréa Dolores Correia Miranda Valdivia, María Luisa Ramos-Ibarra, María José Franco-Barrera, Luis Felipe Arias-Ruiz, Jian M. García-Cruz, Olivia Torres-Bugarín

**Affiliations:** a Researcher Professor, Specialised Dentistry Department, Faculty of Dentistry, Autonomous University of Guadalajara, Guadalajara, Jalisco, México. Study conception and design, collected data, drafted and proofread the manuscript.; b Researcher Professor, Laboratory of Genetic Toxicology, Department of Public Health, Division of Veterinary Sciences, CUCBA, University of Guadalajara, Zapopan, Jalisco, Mexico. Study conception and design, collected data, drafted and proofread the manuscript.; c Professor, Department of Oral and Maxillofacial Surgery, Faculty of Dentistry, Autonomous University of Guadalajara, Guadalajara, Jalisco, México. Study design, drafted the manuscript.; d Student, Laboratory of Histopathology and Molecular Biology, Hospital Ángeles del Carmen, Guadalajara, Jalisco, Mexico. Study design, proofread the manuscript.; e Student, International Medicine Programme of the Autonomous University of Guadalajara. Clerkship Hospital Abrazo Community Health Network, Phoenix, AZ, USA. Study design, drafted the manuscript.; f Researcher Professor, Genotoxic Research Laboratory at School of Medicine, Autonomous University of Guadalajara. Study conception and design, acted as scientific advisor, collected data, drafted the manuscript.; * These authors contributed equally.

**Keywords:** keratocysts, odontogenic cyst, review literature

## Abstract

**Purpose:**

To present updated information on odontogenic keratocyst (OKC) classification, etiology, genetic and molecular alterations, epidemiology, clinical presentation, radiographic characteristics, histological and immune histochemical features, differential diagnosis, treatment, and controversies, as well as a literature review of case frequencies in different countries.

**Materials and Methods:**

Studies were selected using the key words ‘odontogenic keratocyst,’ ‘odontogenic cysts,’ ‘odontogenic keratocyst and clinical study’. Full-text papers were reviewed on the basis of the inclusion and exclusion criteria. The literature search aimed to find articles that would show the frequency of OKC, dentigerous cyst, radicular cyst, and other cysts.

**Results:**

OKC presents local aggression and high recurrence; therefore, a better understanding of its clinical characteristics and the genetic and molecular factors involved in this peculiar and controversial lesion is required. It is always essential to discuss treatment alternatives. Although OKC is an entity with a high recurrence, aggressive treatment is not advisable in all cases because factors such as commitment to anatomical structures and possible complications should be considered. However, periodic radiographic controls are advised.

**Conclusion:**

To reduce the high number of present cases worldwide, it is important to improve knowledge on this pathology so that accurate diagnoses can be achieved and appropriate treatment can be provided. OKC presents local aggression and high recurrence; therefore, a better understanding is needed of the clinical characteristics and genetic and molecular factors involved in OKC. Furthermore, it is always essential to discuss treatment alternatives.

According to the World Health Organization (WHO), odontogenic keratocyst (OKC) is ‘a benign uni- or multicystic intraosseous tumor of odontogenic origin with a characteristic lining of parakeratinized stratified squamous epithelium and has the potential for aggressive, infiltrative behavior.’^54^ OKC is the most common type of odontogenic cyst; it is a noninflammatory developmental tumor, an aggressive benign, odontogenic, cystic neoplasm that occurs intraosseously. Typically, it exhibits slow growth, infiltrative biological behaviour, bone resorption capacity, tooth displacement and a high recurrence rate after surgical treatment.^32^ The characteristic appearance is an expansible, solitary, lucent lesion with a smooth and often scalloped border, most commonly in the posterior mandible surrounding the crown of the third molar; it may displace teeth, causing malocclusion.^43^ The mean age of occurrence is 40 years and arises predominantly in males. There have been case reports of OKCs in other nonosseous locations: most of these involve the gingival tissues, but they can also occur in mucosal, epidermal, and even intramuscular sites.^43,62^ This literature review aims to present updated information on OKC classification, etiology, genetic and molecular alterations, epidemiology, clinical presentation, radiographic characteristics, histological and immune histochemical features, differential diagnosis, treatment, and controversies, as well as a review of case frequencies in different countries.

## Materials and Methods

### Search Strategy

To review the literature, electronic searches were performed in MEDLINE and Google Scholar, using the following key words: ‘odontogenic keratocyst,’ ‘odontogenic cysts,’ ‘odontogenic keratocyst and clinical study.’ First, the literature was searched to find articles that would show the frequency of OKC, dentigerous cyst, radicular cyst, and other cysts. The cut-off period was from 1994 through 2021. English and Spanish language papers were eligible. Second, from each continent, representative items were sought that meet the above characteristics (Table 1).

**Table 1:** Odontogenic cysts in different countries

Year Period	Biopsies		Odontogenic Cysts Type				Country [Reference]
	Total	O. cysts n (%)	OKCs n (%) Place	Dentigerous n (%)	Radicular n (%)	Other n (%)	
**The Americas**							
26	40,000	6879 (17.2)	335 (4.9) 3rd	1662 (24.1)	4468 (65.2)	414 (6.9)	Canada [8]
15	40,568	*ND	*430 (1) -	ND	ND	ND	USA [42]
18	1266	103 (8.1)	34 (33.0) 3rd	58 (56.0)	5 (4.9)	6 (5.9)	SLP, Mex [36]
06	700	*75 (10.7)	30 (40.0) 1st	21 (28.0)	ND	24 (32.0)	Méx, Mex [31]
21	4410	856 (19.4)	184 (21.5) 3rd	283 (33.0)	342 (39.9)	47 (5.6)	Méx, Mex [40]
10	3865	*304 (7.9)	57 (18.7) 3rd	108 (35.5)	ND	139 (45.7)	Méx, Mex [29]
52	23,479	3,939 (16,8)	268 (6.8) 3rd	978 (24.8)	259 (65.9)	99 (2.5)	Brazil [21]
28	ND	2944 (ND)	241(14.2) 3rd	546(18.5)	1494 (50.7)	713 (24.21)	Chile [41]
14	13, 034	1878 (14.4)	520 (27.7) 3rd	522 (27.8)0	754 (40,15)	82 (4.7)	Venezuela [61]
06	109	55 (42.2)	2 (3.6) 3rd	41 (74.5)	12 (21.8)	0	Nicaragua [50]
Total Frequency		17,038	2101 (12.3) 3rd	4219 (24.76) 2nd	9669 (56.74) 1st	1524 (8.9)	
**África**							
15	2190	326 (14.8)	46 (14.1) 3rd	49 (15.0)	222 (68.1)	9 (2.7)	Libyan, North Africa [12]
21	407	*	25 (6.1) 2nd	230 (56.5)	23 (5.7)	ND	South Africa [39]
Total Frequency	2597	604	71 (11.8) 3rd	279 (46.2) 1st	245 (40.6) 2nd	9 (1.5)	
Europa							
14	647	180 (27.8)	3 (0.5) 3rd	61 (9.4)	108 (16.7)	8 (1.2)	Spain [53]
10	--	695 (ND)	133 (19.1) 3rd	154 (22.3)	372 (53.5)	36 (5.1)	Francia [35]
19	12,197	*1318 (10.8)	17 (1.3) 3rd	149 (11.4)	1107 (84.5)	ND	Italy [60]
30	55,446	7121 (12.8)	828 (11.6) 3rd	1292 (18.1)	3724 (52.3)	1277(17.9)	UK [23]
Total Frequency	ND	9314 (ND)	981 (10.5) 3rd	1656 (17.8) 2nd	5311 (57.0) 1st	39 (4.3)	
**Australia**							
58	1305	*ND (18.5)	22 (1.7) 3rd	123 (9.4)	68 (5.2)	ND	Australian [2]
Asia							
10	8563	1283 (17.7)	282 (18.6) 3rd	366 (24)	506 (33.2)	126 (8.4)	Iranian [55]
20	3875	312 (9.0)	32 (36) 2nd	50 (42.5)	27 (19.9)	1 (0.6)	Iranian [38]
10	980	150 (15.3)	12 (8) 2nd	26 (17.3)	73 (48.7)	39 (26)	Indian [26]
12	665	*xref>ND	*565 (84.9)	ND	ND	ND	China [14]
10	739	467 (63.1)	23 (6.8) 3rd	106 (22.7)	230 (49.3)	24 (7.1)	Turkey [20]
9	459	452 (98.5)	64 (13.7)3rd	122 (26.6)	251 (54.7)	7 (1.5)	Turkey [1]
30	127	*112 (88.2)	*75 (66.9) --	ND	ND	ND	Saudi Arabia [2]
10	ND	125 (42)	17 (13.6) 3rd	28 (22.4)	76 (60.8)	4 (0.8)	India [6]
5	252	201 (79.76)	55 (27.4) 2nd	45 (22.39)	101 (50.25)	51 (20.24)	Bangalore, India [47]
14	7117	1252 (17.6)	74 (5.9) 2nd	150 (11.9)	44 (3.5)	286 (21.4)	South Kerala, India [10]
13	413	70 (16.9)	13 (18.6) 2nd	19 (27.2)	1 (1.4)	37 (52.8)	Hamadan, Iran [4]
23	7412	1603 (21.6)	362 (22.6) 3rd	413 (25.8)	563 (35.1)	265 (12.9)	Isfahan, Iran [27]
20	3466	300 (9)	138 (46) 1st	48 (16)	42 (14)	72 (24)	Tehran, Iran [39]
Total Cases	34,068	6327 (18.6)	4340 (68.6)	1373 (1.0)	1914 (39.3)	912 (14.4)	
Place		* 5736	* 3119 (54.4) 1st	* 742 (12.9) 4th	* 784 (13.7) 2nd	* 749 (13.1) 3rd	

ND: no data ND; M: male; O. cysts: odontogenic cysts; OKCs: keratocysts; SLP: San Luis Potosí. Those rows with an asterisk (*) were not included in the total for each continent due to lack of data.

The literature search aimed to determine the following:

1. What is the previous and current classification for odontogenic keratocyst (OKC)?
2. What are the theories related to etiology and genetic factors involved in the OKC?
3. What is the variation in the prevalence of OKCs in different geographic regions and by sex and age?
4. Histological findings, clinical features, and radiographic appearance of OKCs.
5. Differential diagnosis and treatments in the OKC.

### Selection of Studies

In the first screening step, the two reviewers independently evaluated the titles and abstracts of studies retrieved from the electronic search by using the key words. Duplicate papers obtained using different key words were considered only once. The full-text papers were then obtained and reviewed on the basis of the following inclusion and exclusion criteria. Inclusion criteria: clinical studies with follow-up reported, retrospective studies, representative locations of cases on different continents, more recent publication of the same study, and literature reviews. Exclusion criteria: case reports, laboratory studies, studies that did not specify the frequency of keratocysts. Disagreement between reviewers was solved by discussion. If consensus was not reached by the two reviewers, a third author was consulted.

## Results and Discussion

### Classification

OKC was described for the first time by Mikulicz in 1876, but the term OKC was coined by Philisen in 1956. The characteristic features were first described in 1963 by Pindborg. OKC was described by Hansen in 1963 as a well-defined solitary lesion surrounded by a thin cortical layer. Over the years, attempts have been made to understand the nature of OKCs to classify or reclassify them.^5,44^ Some authors may classify OKC as a tumor because of its relatively frequent mitotic activity in the epithelium, presence in chromosomic aberration syndrome, mutation of the PTCH tumor suppressor gene, and association with Gorlin-Goltz syndrome.^45^ However, one of the most debated topics in the 2017 classification was the decision to transfer keratocystic odontogenic tumors (KCOTs) back into the cyst category as OKC, with the evidence put forward for reclassification based on aggressive growth, high recurrence after treatment, and mutations in the PTCH gene.^43^ The WHO reclassified OKC from a benign cyst to a neoplastic lesion (KCOT) in 2005, but this decision was reversed in 2017 because the evidence was not sufficient at the time to reclassify OKC from benign to neoplastic, which is its current classification.^32,62^ Undoubtedly, this decision is very controversial because there are still many researchers who disagree and because it is believed that this will decrease the awareness of the possibility of OKC recurrence and aggressiveness.^56^.

A reversal of the above decision was based on the following evidence:^44^

- Aggressive behavior: OKC is locally destructive and highly recurrent.
- Growth as that of a benign tumor: OKC does not grow by osmotic expansion like other odontogenic cysts, and its unremitting growth is by epithelial proliferation of the wall with folding of the epithelial lining.
- Markers in cyst fluid: the soluble protein content of the cyst fluid is lower than that of other cysts.
- Histopathology and histochemistry: in OKC, the budding basal layer and mitotic figures are frequently found in the suprabasal layers.
- Furthermore, a higher expression of oxidative enzymes (NADH2-, NADPH2-, G6PD, and acid phosphatase), leucine aminopeptidase, and parathyroid hormone has been detected in OKCs than in radicular, residual, and dentigerous cysts. This is attributed to the invasiveness of OKC. Higher levels of PTHrP, which is a protein related to bone resorption, are also observed.^15^

### Etiology

In addition to genetic causes, there are also long-proposed histological links to recurrence, including the presence of daughter cysts, relatively weak folded epithelial lining, and basal cell budding. OKCs originate from the cell rests of the dental lamina (a primitive, embryonic, epithelial, invaginated ridge that forms the tissues of the teeth). During the early developmental stage of dental lamina, it disintegrates into small epithelial clusters and is resorbed. In situations when the clusters are not resorbed, eruption cysts are formed over the developing tooth and delay its eruption into the oral cavity.^24^ Remnants of the dental lamina are sometimes known as the glands of Serres. OKCs are located mainly in the gingival and periodontal ligament, in addition to an extension of the basal cell component of the buccal epithelium. OKC has a 28% to 35% risk of recurrence^32,62^ and can recur in up to 62.5% of cases if not removed completely.^5,57^

### Genetic Factors

The morphogenesis and cytodifferentiation of the dental organs are mediated under the genetic control of regulators such as sonic hedgehog protein (SHH), bone morphogenetic protein, and genes that control the cell cycle, in addition to tumor suppressor genes such as P53 and PTCH, which act as regulators of cell growth.^16,25^ Additionally, it is evident that OKC exhibits dysregulation of the cell cycle and mutations in proliferation regulatory genes, because evidence shows that the mitotic activity in OKC is greater than in other cysts of odontogenic origin. The inactivation of all these genes either by mutation or loss of heterozygosity (LOH) results in the development of tumors. However, the exact genetic mechanisms of the development of OKC lesions are poorly understood.^7^

Kalogirou et al^25^ performed a systematic review and meta-analysis of the immunohistochemical profile of the basal cell of sporadic OKCs. A total of 71 studies were qualitatively analysed, and 61 and 32 markers were evaluated in 1 study and ≥2 studies, respectively. Twenty-five studies reported a differential expression of 29 markers in the form of a higher number of positive cells or greater staining intensity in BCNS-associated OKCs. Meta-analysis for bcl-2, cyclin D1, CD56, CK18, p53, and proliferating cell nuclear antigen (PCNA) showed that none of those markers are distinguishable between BCNS-associated and sporadic OKCs at a 95% confidence interval. They concluded that although several immunohistochemical markers might characterize the OKC phenotype, they cannot discriminate between the BCNS-associated and sporadic OKCs. Coşarcă et al^9^ analysed the immunoexpression of Ki67, p53, MCM3, and PCNA markers in the epithelial remnants of the dental follicles of impacted teeth. They attempted to identify a possible correlation between the immunoexpression of these markers in OKCs to evaluate their evolutionary behavior. The assessment of the four antibodies in the two layers of OKCs shows a positive correlation between Ki67 and MCM3 both for the basal and parabasal layers, with the latter having slightly increased values.

On the other hand, several studies have attempted to identify the expression pattern of protein-coding genes or noncoding transcripts in these lesions to clarify their significance in the pathogenic processes. Moreover, many studies have identified the association between some genetic loci and the susceptibility to the development of these odontogenic lesions.^16^ The p53, PTCH, SHH, and GLI1 genes are the most commonly assessed genes in OKC samples, thus suggesting that these gene signaling pathways play an important role in the mesenchymal epithelium and in cell interactions and proliferation during odontogenic tumor growth and dental development.^34^ The following provide further information on genes associated with OKC samples:

- p53 (tumor protein p53 gene [TP53]) is a tumor suppressor gene that participates in growth arrest, initiates the repair of DNA damage, or induces apoptosis in the G1 phase of the cell cycle. The p53 gene prevents cells from entering the synthesis phase of the damaged cell. If the DNA is repaired, cell cycle arrest is ended. However, if the DNA is not successfully repaired, p53 induces apoptosis and leads the cell to die. In the absence or mutation of p53, DNA damage cannot be repaired, thus leading to the proliferation of damaged cells or to malignancy. The expression of the p53 protein in the cystic epithelium of OKC has not been defined, but the aggressive behavior and high recurrence rate of these lesions might be related to the immunoexpression of this protein. Case studies reported that mutations in the p53 gene are strongly associated with the aggressive behavior and high recurrence of OKC.^24^
- p63, which encodes for TAp63 and ΔNp63 isoforms, is essential for the development of the head and neck region. Several syndromes associated with altered dental development show p63 mutations. Isoforms contain the transcriptional activation domain and is involved in apoptosis and cell proliferation. p63 may act as an oncogene and can be found in the skin, esophagus, oral mucosa and odontogenic epithelium of tooth germ, and dental follicle of impacted teeth.^33^
- PCNA is an essential factor in DNA replication and repair. It is a nuclear protein expressed in mitotic cells, specifically in the late G1 (plays a critical role in initiation of cell proliferation) and S phases of the cell cycle, and is a useful marker for the proliferating fraction of cells in tissue specimens. It forms a homotrimeric ring that embraces the DNA and slides along it, anchoring DNA polymerases and other DNA editing enzymes. It also interacts with regulatory proteins via a sequence motif known as the PCNA-interacting protein box.^18^ In a sample of OKCs, the highest number of PCNA-positive cells was identified in the suprabasal epithelial layer of KCOTs, thus suggesting that these lesions have higher proliferative activity. Furthermore, it was demonstrated that PCNA expression was more pronounced in syndromic KCOTs than in sporadic KCOTs.^59^ PCNA expression was studied in OKCs (n=15) and ameloblastomas (n=46) by using an avidin-biotin-peroxidase complex method on routinely processed paraffin sections. The percentage of PCNA-positive cells determined by point counting was statistically significantly lower in the ameloblastomas (mean: 9.4%, standard deviation [SD]: 11.0) than in OKCs (mean: 29.9%, SD: 24.0). The mean percentage of PCNA-positive cells in the epithelial lining of OKC was not statistically significantly different from those in the peripheral cells of the follicular and plexiform patterns of ameloblastoma. In contrast, OKC exhibited a mean percentage of PCNA-positive cells that was statistically higher than that in other histological elements of ameloblastomas. The present study suggests that OKC is regarded as a benign odontogenic tumor.^48^ On the other hand, OKC showed PCNA expression in all cases (n = 20, 100%); in perapical cysts (n = 10), 60% of cases exhibited PCNA staining.^13^

It is possible that the biological behavior of OKCs may be related to the suprabasal proliferative compartment in the cystic epithelium, as reflected in the high levels of p53, p63, and PCNA. The results indicate that these proteins contribute to the biologic profile of OKCs.^24^ Furthermore, p63 and PCNA immunostaining may represent the immaturity of keratinocytes in OKCs, thus suggesting that these proteins may participate in the regulation of epithelial cell differentiation. The more intense and diffuse expression of p53, p63, and PCNA in OKCs, particularly in the suprabasal cell layer, could help explain the difference in the clinical and pathological behavior of OKCs. This indicates that an abnormal control of cell cycle leads to intrinsic growth potential. Taken together, these data may favor tumorigenesis on OKCs.^24^

- Ki-67 universal proliferation marker is another marker of cell replication. It was significantly more expressed in OKCs than in other types of odontogenic cysts (e.g. radicular cysts and dentigerous cysts),^51^ and its expression was stronger in syndromic lesions than in sporadic lesions.^37^ The increased Ki-67 labeling index, its expression in the suprabasal cell layers of the epithelial lining in OKC, and its correlation with the suprabasal cell layers of the epithelial lining in radicular and dentigerous cysts could contribute to the clinically aggressive behavior of OKC.^51^ In contrast, the use of automated methods and the Aperio Technologies (Vista, CA, USA) computer system did not reveal statistically significant differences in immunoexpression or immunostaining intensities between the two lesions. However, OKC showed a significantly higher cellular proliferation index in the suprabasal layers than in the basal layer. The increased Ki-67 immunoexpression in the suprabasal layers of OKC suggests that it has a different biological behavior and more aggressive proliferation potential than do dentigerous cysts.^19,30^
- PTCH (9q22.3-q31) is part of the patched gene family and indicates LOH in tumor suppressor genes that participates in tumorigenesis. It is an important part of the SHH signaling pathway and encodes the PTCH transmembrane protein, which, together with SMO (smoothened), forms a receptor for SHH ligands and suppresses the SMO-mediated transcription of cellular proliferation genes. Therefore, the lack of PTCH function results in the increased transcription of genes responsible for cell proliferation and, ultimately, tumor formation. The frequency of allelic loss in the 9q22 chromosome is significantly higher in syndromic lesions than in sporadic lesions in OKC. However, after a meta-analysis of the available literature, it was concluded that mutations in the transmembrane are significantly associated with sporadic KCOT. Furthermore, not all OKCs show PTCH1 mutations, and this gene can be inactivated by other mechanisms, such as DNA methylation (to be addressed in the epigenetics section of this paper). Moreover, mutations in other Hh pathway genes, such as SMO, were also described in sporadic OKCs, as well as LOH at the SUFU and PTCH2 loci in sporadic OKCs46.
- BRAF V600E oncogene (7q34) encodes a cytoplasmic serine-threonine kinase. Jain et al^17^ analysed 15 OKC cases and found a complete absence of BRAF V600E mutation in all cases of sporadic OKC and syndromic OKC. However, 6 studies with a total of 103 cases of sporadic OKC evaluated mutations in BRAF V600E. Four of these studies found no mutations, and 2 studies found mutations in 23 reports in total.^22^
- Matrix metalloproteinases (MMPs) are enzymes that play important functions in regulating the integrity and composition of the extracellular matrix (i.e. cell degradation, proliferation, differentiation, and death). MMP1 is one of the major proteases that can degrade type 1 collagen, which maintains the strength and rigidity of connective tissue. MMP1 is associated with the OKC bone matrix and causes the dissemination of this cyst through trabecular spaces. MMP2 resides in the basement membrane of OKC and is involved in the degradation of the extracellular matrix around the cyst.^44^

Methylation patterns in the p16, p21, p27, RB1, and p53 genes of OKC samples, as well as dental follicle and normal mucosa samples, showed that although the methylation pattern of the p16 gene did not differ between the samples mentioned, the p21 gene was methylated in approximately one-third of OKC samples but not in samples from the other groups. The p27 gene was more methylated in dental follicles than in the OKC samples and normal mucosa specimens. RB1 was methylated in two dental-follicle samples.^16^

### Epidemiology

Epidemiological studies are essential because they allow a more accurate establishment of the occurrence of OKC in different populations, thus helping establish a diagnostic hypothesis and plan a biopsy on the basis of clinical and radiographic characteristics. Furthermore, knowledge of the clinical-pathological characteristics recorded in various populations can help identify the possible causes associated with these lesions. The literature indicates a variation in the prevalence of OKCs in different geographic regions^48^ (Table 1), ranging from 0.5% in Spain to 46% in Tehran, Iran, for odontogenic cysts only. As shown in Table 1, when ranking the most frequent cystic lesions (OKCs, dentigerous cysts, and radicular cysts) by continent (the Americas, Europe, Africa, and Asia), OKCs occupied third place.

OKC accounts for approximately 7.8% of all cysts of the jaw, and incidence vary from 4% to 16.5% (Table 2). OKC occurs at all ages, with peak incidence in the second and fourth decade of life. It predominantly occurs in the White population and in males (1.5:1 male:female ratio). In terms of location, it is most commonly seen twice as often in the mandible compared with the maxilla (66.9%). In the mandible, it usually presents in an angle and ascends to the root region (69%-83%).^44,45^ It is usually located in the area of the mandibular third molar and can invade the body and the ascending root.^5^ Keratocysts present as a solitary lesion, except when associated with the Gorlin-Goltz syndrome.^15^ In addition to being characterized by the presence of multiple keratocysts in the maxilla and mandible, this syndrome also presents in the plantar pits, calcification of the falx cerebri, and others.^32^ If the cystic lesion is solitary, without involving retained teeth, differential diagnosis is essential, given the possibility of traumatic bone cyst, inflammatory cyst, lateral periodontal cyst, residual cyst, nasopalatine cyst, central giant cell granuloma, brown hyperparathyroidism tumor, plasmocytomas, vascular malformations, and benign bone tumors.^5^

**Table 2:** Frequency of the most frequent odontogenic cysts by continent

	The Americas	Europa	Africa	Asia	Total
OCKs	1584	904	46	1072	3666
Dentigerous	4090	1507	49	1373	7019
Radicular	9669	4204	220	1914	16,007
Total	15,343	6675	315	4359	26,692

### Clinical and Radiographic Findings

The clinical features and radiographic appearance of OKCs are not characteristics that may lead to misdiagnosis. Clinically, OKC presents as soft tissue swelling and is usually asymptomatic with or without pain, discharge, increase in volume of variable size, coverage by normal mucosa, displacement of teeth, expansion of bone with gross facial asymmetry, and occasionally paresthesia of the lower lip. The expansion of the cyst is very minimal at the initial stage, because of its typical growth in an anterior-posterior direction in the medullar cavity of the bone, thus initially causing obvious bone expansion. In its aggressive growth phase, it can produce bone deformities, invade adjacent tissues, expand cortically, and move teeth.^5,32,44^

The radiographic presentation of OKCs is variable, and the radiographic appearance of OKC may range from a small unilocular to a large, multilocular, well-defined radiolucency, which shows extensive involvement with little or no bony expansion. Approximately half of all OKCs occur at the angle and root of the mandible, are surrounded by smooth or scalloped margins usually with sclerotic borders, can sometimes displace surrounding teeth, and rarely promote radicular resorption. Nevertheless, other odontogenic lesions may show similar radiological findings. For OKC, the final diagnosis depends on histological examination.^24^ In general, it is detected by routine radiographic findings because it is asymptomatic in its early stages.^5^

### Histological Features

Histologically, OKCs arise from the dental lamina and consist of a regular thin cystic cavity containing desquamated keratin. The cyst is lined with a uniform parakeratinized or orthokeratinized squamous stratified epithelium of 6-10 polarized layers of cuboidal cells, thick with higher proliferative activity. The mitotic activity in OKC is higher than other cysts of odontogenic origin and tends to recur and form compartments. It also has structural alterations in the capsule, with a distinct, tall, columnar basal cell layer resembling a palisade, with hyperchromatic nuclei, which tend to be vertically oriented.^24,32^ The infiltrative growth with the adjacent connective tissue is normally flat, with a potential for budding of the basal layer and formation of small satellite or daughter cysts.^24^ Pigmented odontogenic lesions are rare; in these lesions, melanin is observed within the cytoplasm of the cystic lining epithelium or tumor cells.^49^ The expression of high epidermal growth factor receptor in OKC has supported its intrinsic growth potential, which is not present in other odontogenic cysts.^32^ OKC tends to grow along spongy canals with little cortical expansion. This is probably due to intraluminal hyperosmolality and active epithelial proliferation, in addition to the collagenolytic activity of the cyst wall and the synthesis of interleukin 1 (IL-1) and interleukin 6 (IL-6) by keratinocytes.^57^

#### Histological features for the diagnosis of OKC^5,49^

- The name ‘OKC’ refers to the fact that keratin is produced by the cystic lining. It is a parakeratin-lined cyst-like lesion within bone. OKC is the one of the rare and distinctive developmental odontogenic cysts from the dental lamina and contains clear fluid and a cheesy material resembling keratin debris.
- Thin, parakeratinized (85%–95%), orthokeratinized, stratified squamous epithelium lining with a ribbon-like appearance that is typically 8-10 uniform-layers thick.
- Lack of rete ridges/pegs
- Surface keratinization is corrugated and rippled and is mostly parakeratosis (keratinized cells with nuclei)
- Well-defined basal cell layer having cuboidal or columnar cells with hyperchromatic nuclei arranged such that they appear as a ‘picket fence’ or ‘tombstones’
- The cyst lumen contains traces of keratin and satellite cysts in the connective tissue
- A thin, spinous cell layer often showing direct transition from basal cell layer (artifact separation of epithelium from basement membrane) and intracellular edema of the spinous cell layer
- Cystic wall composed of thin, usually non-infamed fibrous connective tissue
- Satellite cysts, daughter cysts (7%–30%), solid epithelial proliferation, and residual odontogenic basal layer budding. The fibrous connective tissue wall may become mineralized and include cholesterol crystals and Rushton bodies.

### Differential Diagnosis

The diagnosis of OKC is primarily based on histopathological features. It typically shows a thin, friable wall, which is often difficult to enucleate from the bone in one piece. The cystic lumen may consist of a clear liquid, similar to a transudate of serum, or a cheesy material. The thin fibrous wall is essentially lacking an inflammatory infiltrate. Small satellite cysts, cords, or islands of odontogenic epithelium are sometimes seen within the fibrous wall.^28,57^ Histological characteristics include myxoma, ameloblastoma, central giant cell granuloma, and odontogenic cysts. Radiographically, these findings in OKC may resemble a dentigerous cyst (40%), residual cyst, redicular cyst, lateral periodontal cyst (25%), primordial cyst (25%), globulomaxillary cyst (10%), unicystic ameloblastoma, A-V malformation, or a fibro-osseous lesion at the initial stages. OKC in the anterior midline maxillary region can be mistaken for nasopalatine duct cysts. Peripheral OKC within the gingival soft tissues is rarely reported.^21^

### Treatment

Owing to OKC’s aggressive potential, it requires thorough surgical treatment and long-term behavioral controls.^5^ Treatment is still controversial because there are different approaches – all of them with advantages and disadvantages. These treatment modalities include conservative methods, such as simple enucleation with primary closure, enucleation with open packing, decompression, or marsupialization, more aggressive techniques using cryosurgery (with liquid nitrogen), chemical destruction via application of Carnoy’s solution (which previously consisted of a mixture of absolute alcohol, chloroform, glacial acetic acid, and ferric chloride; chloroform is no longer used because of its carcinogenicity); and radical surgical techniques with bone resection. The literature shows a lack of consensus on a uniform treatment plan for OKCs and does not provide adequate evidence for determining which modality is the most effective in lowering morbidity or preventing recurrence. Decompression of odontogenic cystic lesions has been widely used as a more conservative treatment; it requires a much smaller window by creating an opening into the cystic cavity and suturing a cylindrical device to its periphery. This technique was used by Tucker in 1972, who was the first to describe the use of decompression and secondary enucleation as a treatment option for OKC.^36-38^ Therefore, the current treatment for keratocysts is divided into 1) conservative treatment, 2) radical or aggressive treatment, and 3) adjuvant treatments.^52^

#### 1) Conservative treatment

- Marsupialization: A permeable tubular device is placed inside the cyst for periodic instillations. This method aims to reduce the initial pressure and induce bone formation, thus reducing the size of the lesion, in addition to eliminating the involvement of anatomical structures (such as the lower dental nerve, maxillary sinus, nasal cavity, and mandibular or and maxillary bone border).^52^
- Enucleation: This is performed after marsupialization or as a first option and consists of the total removal of the lesion.

#### 2) Radical or aggressive treatment

- Involves complete or partial resection (mandibulectomy or maxillectomy)^3^

#### 3) Adjuvant treatment

- Chemical (Carboy’s solution): Carnoy’s solution consists of 60% ethanol, 30% chloroform, 10% acetic acid, and 0.1 g ferric chloride. Its mechanism of action is described as the coagulation of proteins and the prevention of recurrence of the lesion. It is applied on the bone margin after the enucleation of the lesion. It has been shown that penetration into the tissues is 1.54 mm deep. After 5 min of application, it causes chemical cauterization owing to its impregnation capacity, devitalization, and cystic cell fixation.^11^
- Physical (liquid nitrogen): Tissues are devitalized beyond the visible margins of the lesion at a low temperature (20°C) by using liquid nitrogen. This method fosters the formation of new bone, with the possibility of immediately placing a bone graft to accelerate bone healing and reduce the risk of pathological fracture. However, it is difficult to apply safely, and can damage soft and hard tissues owing to low precision.^57^
- Mechanical (peripheral osteotomy): Peripheral osteotomy mechanically removes the epithelium and the surrounding cells corresponding to the cyst after enucleation.^56^

A search was made of articles from different countries from different continents that considered OKC in their analysis. In general, the literature described OKC to be among the globally most commonly encountered odontogenic entities of all odontogenic cysts. But if these entities are analyzed individually for each country, the OKCs are 3rd most common, because the analysis of odontogenic tumors frequently does not consider OKCs (Table 2).

## Conclusions

To reduce the high number of OKC cases worldwide, information on different aspects of OKC must be disseminated to improve diagnostic accuracy and facilitate the provision of appropriate treatment. OKC presents local aggression and high recurrence; therefore, a better understanding of its clinical characteristics and the genetic and molecular factors involved in this peculiar and controversial lesion is required. It is always essential to discuss treatment alternatives. Although OKC is an entity with high recurrence, aggressive treatment is not advisable in all cases, because factors such as involvement of anatomical structures and possible complications should be considered. However, periodic radiographic controls are advisable.
